# Novel device therapies in heart failure: focus on patient selection

**DOI:** 10.3389/fcvm.2025.1419873

**Published:** 2025-02-25

**Authors:** Amrita Balgobind, Daniel Asemota, Emily Rodriguez, Phuuwadith Wattanachayakul, Marat Fudim, Miguel Alvarez Villela

**Affiliations:** ^1^ Division of Cardiology, Department of Internal Medicine, Montefiore Medical Center/Albert Einstein College of Medicine, Bronx, NY, United States; ^2^ Department of Cardiology, Lenox Hill Hospital, Northwell Health, New York, NY, United States; ^3^ Department of Medicine, Lenox Hill Hospital, Northwell Health, New York, NY, United States; ^4^ Department of Medicine, Jefferson Einstein Hospital, Philadelphia, PA, United States; ^5^ Sidney Kimmel Medical College, Thomas Jefferson University, Philadelphia, PA, United States; ^6^ Division of Cardiology, Department of Internal Medicine, Duke University School of Medicine, Durham, NC, United States; ^7^ Duke Clinical Research Institute, Durham, NC, United States

**Keywords:** device therapy, heart failure, baroflex activation therapy, cardiac contractility modulation, Mitraclip, AccuCinch

## Abstract

The increasing prevalence of heart failure (HF) has led to advancements in therapeutic strategies, including the development of new pharmacological treatments and the expansion of guideline recommendations across the spectrum of left ventricular ejection fractions. Despite these advancements, the full benefits of guideline-directed medical therapy (GDMT) are often limited by various barriers that result in incomplete implementation or suboptimal responses. For patients who cannot tolerate or only partially respond to GDMT, therapeutic options remain limited. This gap is particularly significant for those with contraindications to heart replacement therapies (HRT), such as left ventricular assist device (LVAD) or heart transplant. In light of these potential limitations, this review article proposes categorizing HF patients into four distinct phenoprofiles based on their tolerance to GDMT and candidacy for HRT. Considering these HF phenoprofiles may guide treatment decisions regarding the selection and use of novel device-based HF therapies. Furthermore, we summarize data on commercially available and emerging device-based HF therapies, evaluating their clinical utility, mechanisms of action, and selection criteria based on current evidence. Finally, we describe clinical cases across various proposed HF phenoprofiles to illustrate how these HF profiles can guide the use of novel device-based therapies to achieve clinical stability, improve GDMT tolerance, or serve as a bridge to, or be used in tandem with HRT in select patients.

## Introduction

The prevalence of heart failure (HF) is rising among the aging population, accompanied by an increasing burden of comorbidities (1). In the United States, HF prevalence is projected to grow by 37% between 2015 and 2030, with a 57% increase among those aged 65 and older (2). This trend is associated with a significant increase in adverse outcomes, including higher mortality rates and more frequent rehospitalizations (3). As HF becomes more prevalent, advancements in therapeutic strategies have emerged, including new pharmacological treatments and expanded guideline recommendations across the spectrum of left ventricular ejection fractions (4).

Despite these advancements, significant treatment gaps persist. In heart failure with preserved ejection fraction (HFpEF), effective treatment options remain limited, with only diuretics receiving a Class I recommendation in the 2022 American College of Cardiology (ACC)/American Heart Association (AHA)/Heart Failure Society of America (HFSA) Guidelines for the Management of Heart Failure and with sodium–glucose cotransporter-2 inhibitors (SGLT2i) being the only other drug class to demonstrate effectiveness in randomized clinical trials (5). In contrast, heart failure with reduced ejection fraction (HFrEF) has a stronger evidence base, with several drug classes earning Class I recommendations in the 2021 European Society of Cardiology (ESC) guidelines and 2022 ACC/AHA/HFSA guidelines (5, 6). However, real-world management of HFrEF continues to face challenges, particularly due to either incomplete implementation of guideline-directed medical therapy (GDMT) or suboptimal patient response.

Awareness of these limitations coupled with important changes in regulations in the United States have led to the development of novel device-based therapies for HF patients. The FDA Breakthrough Device Program has been instrumental in allowing early market access to new devices while linking them with improved reimbursement from the Centers for Medicare & Medicaid Services (7).

In this evolving landscape, clinicians must integrate these emerging technologies into existing patient management strategies. Current approaches prioritize escalating GDMT to the maximum tolerated doses, followed by assessing patients for device-based therapies, such as cardiac resynchronization therapy (CRT), and valvular interventions, such as MitraClip and TriClip where applicable (4). Additionally, autonomic, and electrophysiological modulation therapies, including baroreflex activation therapy (BAT), vagus nerve stimulation, and cardiac contractility modulation (CCM), are considered based on their specific advantages in targeting the underlying pathophysiological or anatomical abnormalities in HF. Select patients remain symptomatic despite the implementation of GDMT and utilization of these devices, or are not eligible for established technologies. In this population, the strategy often shifts to watchful waiting with heart replacement therapies (HRT) such as LVADs or heart transplants becoming the focus if and when the patient's clinical condition worsens (8).

In this review, we explore the potential limitations of the current HF treatment paradigm and propose a novel approach to categorize patients into distinct phenoprofiles based on their response to GDMT and candidacy for HRT. We also summarize current data on device-based therapies and demonstrate how these technologies can be applied to the different HF phenoprofiles through real-life and hypothetical clinical cases, potentially offering alternative therapeutic options for patients who remain symptomatic after standard therapies.

### Potential limitations in GDMT

#### Incomplete implementation of GDMT

Despite strong evidence for improved outcomes with the implementation of combination medical therapy in HF patients, the proportion of patients achieving target doses of the four pillars of GDMT, including beta-blockers, angiotensin-converting enzyme inhibitors/angiotensin II receptor blockers/angiotensin receptor neprilysin inhibitors (ACEI/ARB/ARNI), mineralocorticoid receptor antagonists (MRA), and SGLT2i, remains low in contemporary registries.

For instance, in the Registry to Improve the Use of Evidence-Based Heart Failure Therapies in the Outpatient Setting (IMPROVE-HF), increases in GDMT dosing were modest, even with a structured intervention aimed at reaching maximally tolerated GDMT dosages over 24 months (9). Similarly, in the subsequent data on the Change the Management of Patients with Heart Failure (CHAMP-HF) registry, the proportion of patients without contraindications for GDMT who reached target doses was lower than reported in clinical trials and further decreased over the 12-month follow-up period (10). Disease progression and medication intolerance were frequently cited as reasons for dose reductions or discontinuation in this cohort, highlighting the challenges in maintaining optimal therapy in real-world settings.

Additionally, chronic kidney disease (CKD) has become a significant factor that prevents GDMT maximization. Although CKD is a crucial risk modifier in HF, patients with advanced CKD were excluded from the Prospective Comparison of ARNI with ACEI to Determine Impact on Global Mortality and Morbidity in Heart Failure (PARADIGM-HF) trial and are underrepresented in the CHAMP-HF and other contemporary registries (10–12). However, real-world observational studies of patients with HFrEF showed that approximately 26% of patients have CKD Stage 3 or higher (GFR < 60 ml/min/1.73 m²), and these patients face increasing mortality with progression of renal disease (13).

#### Incomplete response to GDMT

Among patients receiving target doses of GDMT, a significant yet undefined proportion exhibit an incomplete response to GDMT and remain severely symptomatic. These patients remain at high risk for frequent heart failure hospitalization (HFH) and increased cardiovascular mortality (14). Over time, they often progress to advanced stages of the disease, facing a poor short-term prognosis. To address this challenge, scoring systems such as the MAGGIC score have been developed to help clinicians identify high-risk patients, preventing “therapeutic inertia” and raising awareness for the need for additional therapies that may extend beyond GDMT and established device-based HF interventions (15).

Established device-based therapies with proven efficacy in HF, such as CRT and MitraClip, exist as options for patients with an incomplete response or inability to tolerate GDMT (7). However, only 30%–40% of HFrEF patients who remain symptomatic on GDMT are eligible for CRT based on current criteria, and nearly 30% of those who receive CRT are considered non-responders (16). MitraClip, meanwhile, is suitable for a subset of patients with severe “disproportionate” mitral regurgitation and HFrEF, provided they have the appropriate anatomy for (TEER) transcatheter edge-to-edge repair (5).

For patients who progress to advanced HF, HRT such as LVAD or heart transplantation is a potential option. Yet, these life-saving therapies may be contraindicated in up to 50% of patients in the setting of advanced age or significant comorbidities (17). This highlights the urgent need for alternative therapeutic approaches for this high-risk population, as many patients may be ineligible for commercially effective treatments currently available. As a result, significant gaps remain in the current HF management paradigm, leaving clinicians with limited options for patients with advanced CKD, advanced age, frailty, or incomplete response to medical therapy who continue to experience recurrent HFH or severe impairment in functional capacity.

### Contemporary HF patient phenoprofiles

**Figure 1:**
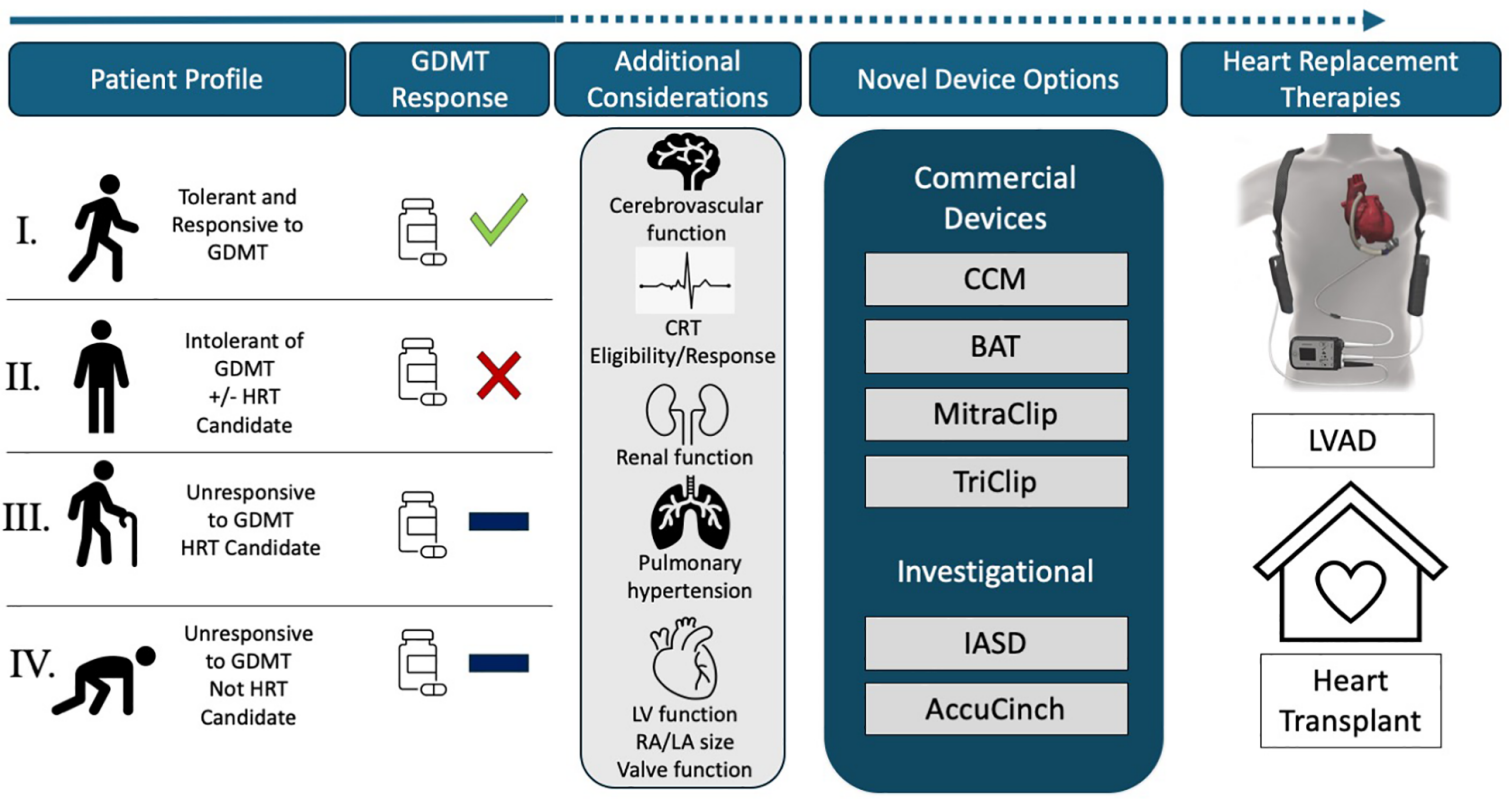
Patient phenoprofiles according to GDMT response and other therapeutic options.

Based on the response to established GDMT and eligibility for HRT, we can postulate that there are four distinct patient phenoprofiles in the current HF landscape, as illustrated in Figure 1:

1. Phenoprofile I: Those who are *responsive to medical therapy* in whom target doses of GDMT are achieved or have not been achieved due to non-medical reasons
2. Phenoprofile II**:** Those with *medical intolerance* to GDMT due to hypotension, advanced chronic kidney disease, or other reasons *independent of candidacy for heart replacement therapies*
3. Phenoprofile III: Those who tolerate target doses of GDMT but have *incomplete response* and are *potential candidates for heart replacement therapies* currently or in the future
4. Phenoprofile IV: Those who tolerate maximal GDMT but have *incomplete response* and *are not candidates for heart replacement therapies* due to *major contraindications*

Patients in Phenoprofile I should continue GDMT treatment as long as they show a positive response with symptomatic improvement. For those receiving suboptimal dosing, achieving target levels can be facilitated through structured GDMT implementation programs and financial assistance.

For patients in Phenoprofiles II, III, and IV, we propose that device-based therapies play a crucial role in their treatment, addressing the gaps that remain after the initiation of standard medical therapies. For patients in Phenoprofile II, novel device-based therapies can enhance functional capacity and quality of life while reducing the risk of HFH, even in the absence of GDMT. In patients with Phenoprofile III, these devices can help improve overall survival, working in tandem with HRT and challenging the current “watchful waiting” approach often adopted with these patients.

Meanwhile, for patients in Phenoprofile IV, device-based therapies can play several critical roles. First, they can act as a bridge to candidacy for HRT by aiding in physical rehabilitation, weight loss, or preparation for necessary future medical procedures. Additionally, these therapies can improve the quality of life when other treatment options are unavailable. Lastly, they may serve as an adjuvant to enhance GDMT tolerance, helping patients manage their condition more effectively despite existing limitations.

### Commercially available device-based HF therapies

#### Cardiac contractility modulation (CCM)

**Figure 2:**
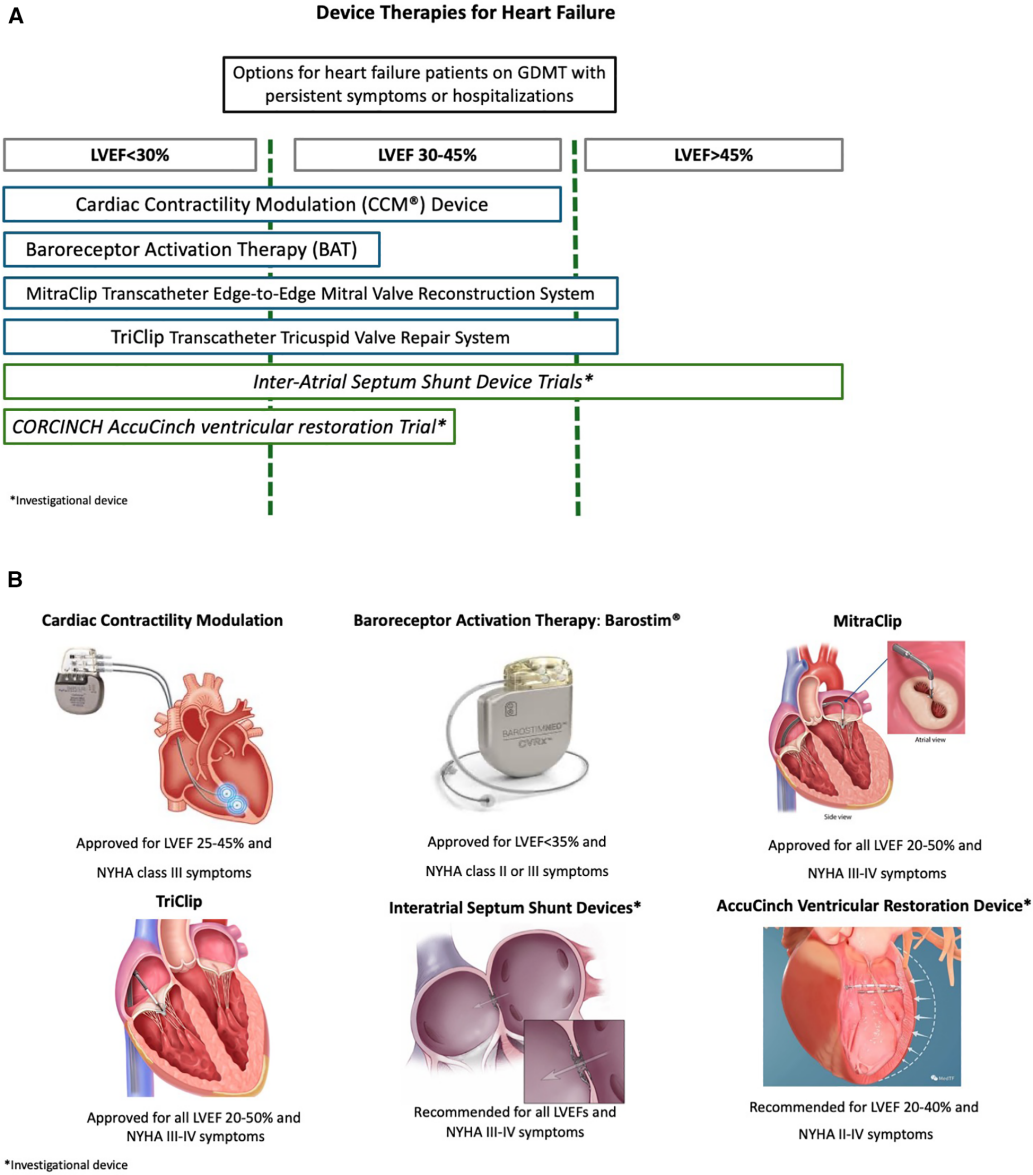
**(A,B)** Commercially available device-based HF therapies.

CCM therapy, delivered via the FDA-approved Optimizer III device (Impulse Dynamics, Orangeburg, NY, USA), uses biphasic, long-duration, high-voltage electrical signals applied to the right ventricular septum during the absolute refractory period (18). Figure 2 demonstrates the commercially available device-based therapies for HF. Since these electrical signals are released during the absolute refractory period, they do not directly cause myocardial contraction but enhance ventricular contractility by triggering acute and chronic cellular changes, promoting favorable myocardial remodeling without increasing oxygen demand (19). The early effects of CCM therapy include increased phosphorylation of troponin and myosin-binding protein C, leading to a positive inotropic effect (20). Over time, CCM therapy also reverts maladaptive gene expression, which may ultimately reverse left ventricular pathological remodeling in patients with HFrEF. CCM has been extensively studied in patients with HFrEF with a recent meta-analysis of four randomized controlled trials demonstrating significant improvements in key outcomes such as peak oxygen consumption, 6 min walk test distance, and quality of life as measured by the Minnesota Living with Heart Failure Questionnaire (MLWHFQ) (21). CCM may also have a role in patients with graft failure after heart transplantation with case reports demonstrating its use in patients with refractory heart failure after transplant (22).

Generally implanted in the right chest, the CCM therapy is similar in size to a dual-chamber pacemaker and is often chosen for patients who already have an implantable cardioverter defibrillator (ICD). The device requires weekly recharging and has an estimated lifespan of 15–20 years before replacement is necessary (18). It is compatible with various commercially available ventricular pacing leads, giving the implanter flexibility in choosing the most suitable option. Additionally, the CCM therapy is FDA-approved as MRI-conditional, ensuring that patients can safely undergo MRI scans when needed (22). Currently, CCM therapy is FDA-approved for HFrEF patients with NYHA Class III or IV symptoms and LVEF 25%–45%, who remain symptomatic despite optimal GDMT and are ineligible for or non-responsive to CRT (7).

#### Baroreflex activation therapy (BAT)

BAT, delivered via the Barostim Neo System (CVRx, Minneapolis, MN, USA), is a form of autonomic modulation that works by electrically stimulating the carotid baroreceptor (23). This is achieved through a surgically implanted lead placed over the carotid sinus, which is connected to a pulse generator implanted subcutaneously in the chest. After implantation, the device's electric pulse amplitude is gradually increased over a 3-month period during follow-up office visits using an external programmer. In clinical trials, typical settings included an 8.7 mA amplitude, 125 μs duration, and 40 pps frequency (24). These electrical stimuli work overtime to decrease sympathetic activity and increase parasympathetic activity, effectively alleviating heart failure symptoms (7).

A meta-analysis of four trials, including both experimental and control cohorts, showed that BAT significantly improved outcomes including LVEF, MLWHFQ scores, and 6 min walk test distances compared to GDMT treatment (25). The analysis also demonstrated reductions in left ventricular end-diastolic volume (LVEDV) and diastolic blood pressure. However, in terms of long-term cardiovascular outcomes, the Baroreflex Activation Therapy for Heart Failure (BeAT-HF) trial found no significant difference in the composite endpoint of cardiovascular mortality and HF morbidity between the BAT and control groups (rate ratio 0.94, 95% CI 0.57–1.57; *p* = 0.82) (23). Despite these findings, BAT remains approved for symptomatic improvement in HFrEF.

Key features of BAT therapy include no need for transcutaneous charging and a battery life of 25–100 months, with the possibility of a pulse generator exchange at the end of service. It is FDA-approved as MRI-conditional, ensuring MRI scans can be performed safely. BAT is currently approved for HFrEF patients in NYHA Class III (or Class II with recent Class III history within the last 3 months), with an LVEF of 35% or less, an NT-proBNP level below 1,600 pg/ml, and no Class I indication for CRT (25).

#### MitraClip

The MitraClip Transcatheter Edge-to-Edge Mitral Valve Reconstruction System (Abbott, Santa Clara, CA, USA) is the first device for transcatheter edge-to-edge mitral valve repair, providing a new treatment option for patients with HF and secondary MR (26). In patients with HFrEF, secondary MR is relatively common, and optimal GDMT should be prioritized. After 3–6 months of optimized medical therapy, clinical revaluation is crucial to assess the need for mitral valve intervention (5). Data from the Cardiovascular Outcomes Assessment of the MitraClip Percutaneous Therapy for Heart Failure Patients with Functional Mitral Regurgitation (COAPT) and Percutaneous Repair with the MitraClip Device for Severe Functional/Secondary Mitral Regurgitation (MITRA-FR) trials, which compared the efficacy of MitraClip vs. GDMT for the treatment of secondary MR from LV systolic dysfunction, have provided important insights (27, 28). The COAPT trial, which involved 614 HF patients with an LVEF of 20%–50% and moderate-to-severe secondary MR despite optimal GDMT, demonstrated that MitraClip significantly reduced HFH, lowered all-cause mortality, and improved quality of life (27). In contrast, the MITRA-FR trial, which included 304 HF patients with moderate-to-severe secondary MR and an LVEF of 15%–40%, found no significant difference in all-cause mortality between the MitraClip and control groups (28).

The differences in clinical outcomes are significant and may be attributed to variations in baseline characteristics between the trials. The COAPT trial enrolled HF patients with “disproportionate MR,” characterized by smaller LV end-diastolic volumes, and more severe MR (7, 26). This distinction has led to the development of the concepts of “severe and disproportionate MR” which describe the relationship between MR severity and LV remodeling. These findings underscore the importance of careful patient selection, as the COAPT trial specifically targeted patients with severe and disproportionate MR, all of whom exhibited significant LV remodeling after mitral valve intervention. MitraClip is currently recommended for HF patients with an LVEF of 20%–50% and severe secondary MR who continue to experience symptoms despite GDMT (5). Candidates should also have suitable mitral valve anatomy, along with an LV end-systolic dimension of 70 mm or less and a pulmonary artery systolic pressure of <70 mmHg.

#### TriClip

The TriClip Transcatheter Tricuspid Valve Repair System (Abbott, Santa Clara, CA, USA) is a transcatheter edge-to-edge repair device specifically designed for treating tricuspid regurgitation (TR), modeled after the MitraClip device used for mitral valve repair (29). A key study providing evidence for its effectiveness is The Trial to Evaluate Cardiovascular Outcomes in Patients Treated with the Tricuspid Valve Repair System Pivotal (TRILUMINATE Pivotal; NCT 03904147), which was a major international, randomized, controlled trial (30). This trial involved 350 patients with severe symptomatic TR who were on optimized GDMT, with exclusions for those with pulmonary artery systolic pressure >70 mmHg, precapillary pulmonary hypertension, LVEF <20%, or unsuitable tricuspid valve anatomy. The results were promising, showing that the TriClip significantly improved a hierarchical composite outcome, which included all-cause mortality, tricuspid valve surgery, heart failure hospitalizations, and enhanced quality of life at 1 year.

Building on these findings, the study, An Observational Real-World Study Evaluating Severe Tricuspid Regurgitation Patients with the Abbott TriClip Device (bRIGHT: NCT 04483089), provided further insights by examining the device's performance in a real-world post-market setting (31). This study involved a larger and more diverse group of 511 patients with more severe TR and complex valvular anatomies. Despite the increased severity of TR and complexity of cases, the TriClip continued to show positive outcomes, including a reduction in TR severity, improved symptoms, and enhanced quality of life. As of the latest evidence, the US FDA approved the TriClip in April 2024 for the treatment of symptomatic severe TR in patients on optimized medical management. This approval marks a significant advancement, providing a minimally invasive option to restore valve function without the need for high-risk open-heart surgery (32).

#### Phrenic nerve stimulation (PNS)

The phrenic nerve stimulation remedē System (Respicardia Inc., Minnetonka, MN, USA) is designed to treat central sleep apnea (CSA), a condition characterized by temporary brainstem-driven respiratory drive loss, leading to episodes of apnea or hypopnea (33). This respiratory disruption, often triggered by heightened instability and an exaggerated response to PaCO_2_ fluctuations, is closely linked to HF, affecting up to 40% of HFrEF patients and 20% of HFpEF patients (34).

While non-invasive ventilatory support, such as continuous positive airway pressure, has proven ineffective for CSA treatment, PNS has emerged as a promising alternative (35). In the remedē System Pivotal Trial, which included 151 CSA patients, 64% with comorbid HF, the PNS treatment group demonstrated a significant reduction in the apnea–hypopnea index (AHI) by 50% or more at 6 months, alongside improvements in quality of life and oxygenation (36). Notably, 91% of PNS subjects remained free of serious adverse events after 1 year, underscoring its favorable safety profile. As of October 2017, the PNS remedē System has been FDA-approved as a treatment option for moderate-to-severe CSA.

#### Implantable pulmonary artery pressure monitoring devices

##### CardioMEMS HF System

The CardioMEMS HF System (Abbott, Sylmar, CA, USA) and Cordella PA device (Endotronix, Inc., Chicago, IL, USA) are available for remote pulmonary artery pressure (PAP) monitoring in HF (37). Emerging data highlight the clinical importance of hemodynamic congestion, which involves early changes in cardiac filling pressures and other upstream physiological parameters that can be detected days or weeks before clinical HF decompensation (38). Monitoring PAP serves as an early marker for impending heart failure-related hospitalizations, with reductions in PAP linked to fewer hospitalizations, regardless of LVEF (39).

The CardioMEMS HF System is an implanted wireless pressure sensor in the left pulmonary artery that continuously measures systolic, diastolic, mean PAP, and pulse rate (40). Patients use a CardioMEMS pillow to remotely collect and automatically transmit this data to a secure database for review. This system allows for the monitoring of cardiac filling pressures in real time, allowing for timely HF treatment adjustments. By tracking these pressures, clinicians can intervene early to prevent decompensation, optimize management, and potentially reduce HFH. As of current evidence, a recent meta-analysis of three pivotal randomized controlled trials—CHAMPION, GUIDE-HF, and MONITOR-HF—examined 1,898 ambulatory heart failure patients in NYHA Classes II–IV. These patients had either been hospitalized for heart failure within the past 12 months or had elevated plasma NT-proBNP levels (37). The analysis demonstrated that PA pressure-guided heart failure management significantly improved outcomes, with a composite reduction in total HFH, urgent visits, and all-cause mortality, yielding a pooled hazard ratio (HR) of 0.75 (95% CI 0.61–0.91, *p* = 0.004). Although the trend toward reduced all-cause mortality was observed, the pooled analysis did not reach statistical significance, with a pooled HR of 0.92 (95% CI 0.73–1.16, *p* = 0.495).

##### Cordella PA device

The Cordella PA device is another remote PAP monitoring device with a wireless pressure sensor implanted in the right pulmonary artery. Patients must use a wireless handheld reader, which collects and transmits the data to a secure cloud-based management portal. The clinical team then reviews this information, enabling timely interventions based on real-time hemodynamic data (7). The potential of the Cordella PA sensor in managing HF was first studied in the SIRONA and SIRONA II trials, demonstrating the device's safety, accuracy, and feasibility (41). Furthermore, the PROACTIVE-HF study further explored the effectiveness of the Cordella PA sensor. This study involved 456 patients with heart failure who exhibited NYHA Class III symptoms, recent HFH, and/or elevated NT-proBNP levels (42). The study initially followed a randomized, single-blind design and later transitioned to a single-arm, open-label format with blinded assessment, focusing on pre-specified safety and effectiveness endpoints over 6 months. The data revealed promising outcomes for the Cordella PA device, significantly reducing HFH and all-cause mortality and surpassing performance goals (43). Additionally, patients using the sensor experienced improvements in quality of life, functional capacity, and NT-proBNP levels.

### Emerging device-based HF therapies

#### AccuCinch transcatheter left ventricular restoration system

The AccuCinch Transcatheter Left Ventricular Restoration (TLVR) System (Ancora Heart, Santa Clara, CA, USA) is a device designed for patients with HFrEF and a dilated left ventricle (LV). This system is delivered via a transfemoral approach, retrogradely accessing the LV through the aortic valve under fluoroscopic and echocardiographic guidance (44). The procedure involves placing a series of anchors on the inner surface of the LV just below the mitral annulus. Once positioned, these anchors are cinched to reduce the size and reshape the LV, thereby decreasing wall stress and promoting reverse remodeling.

A pivotal multicenter trial, “Clinical Evaluation of the AccuCinch Ventricular Restoration System in Patients With Symptomatic Heart Failure With Reduced Ejection Fraction” (CORCINCH-HF; NCT04331769), assessed the effectiveness of the AccuCinch system in HFrEF patients (45, 46). The trial includes participants with an LV end-diastolic diameter of 5.5 cm, who are stable on maximally tolerated GDMT, who have moderate or less MR, and who have no severe CKD. This study compares the outcomes of AccuCinch placement with standard medication therapy, with early results indicating promising decreases in LV volume and improvements in quality of life and exercise endurance.

#### Transcatheter interatrial shunt creation

The Corvia interatrial shunt device (Corvia Medical Inc., Tewksbury, MA, USA) and the V-Wave system (V-Wave, Caesarea, Israel) are devices designed to create interatrial communication by being implanted in the atrial septum. These technologies aim to decrease left atrial pressures by dynamically shunting blood to the right atrium during exercise, thereby reducing dyspnea (47). They have been proposed as potential therapies for both HFrEF and HFpEF.

To date, these interatrial shunt devices have been studied in only two randomized controlled trials, both of which showed neutral overall results. Regarding the Corvia interatrial shunt device, the REDUCE LAP-HF II study (NCT03088033) was a multicenter, open-label, non-randomized trial involving 626 patients with HFpEF and HFmrEF (48). This study found that the Corvia interatrial shunt device provided no added benefit in reducing the total rate of HF events or improving patient health status.

As for the V-Wave system, the RELIEVE-HF trial (NCT03499236) was a prospective, multicenter, randomized study that included 508 patients with mixed HFrEF and HFpEF, comparing shunt implantation with a sham procedure (49). The overall results of this trial were neutral, showing no benefit of the shunt on mortality, HF events, or quality of life. However, a pre-specified analysis revealed a harm signal in the HFpEF cohort and a strong benefit signal in patients with HFrEF, findings that could help serve as the basis for future studies.

### Case studies in device-based HF therapies: emphasizing patient selection

In the following section, we present a series of real-world patient cases from our clinical practice, alongside hypothetical scenarios with distinct HF phenoprofiles based on their response to GDMT and eligibility for HRT. These cases illustrate the application of various novel devices in different clinical contexts.

#### Case 1 Phenoprofile II: GDMT intolerance due to advanced CKD—managing recurrent HF hospitalizations with CCM therapy

##### Clinical scenario

A 74-year-old male with a history of prior thoracic aortic aneurysm repair, transfusion-dependent monoclonal gammopathy of undetermined significance (MGUS), and stage 3b CKD, presented with acute decompensated heart failure (ADHF). His LVEF was 40%, with an LVEDD of 5.7 cm, and he had moderate MR. After several HFH, he was referred to the HF clinic for management. Despite high-dose loop diuretics (torsemide 60 mg twice daily), euvolemia could not be maintained. Attempts to initiate ARNI resulted in worsening of his renal function with a creatinine increase from 2.5 to 2.5 mg/dl and hyperkalemia with a serum potassium of 5.6 mEq/L. He required a new hospitalization with admission to the cardiac intensive care unit requiring a continuous infusion of bumetanide to achieve euvolemia.

##### HF device therapy selected

In this patient with HFmrEF with recurrent HFH and advanced CKD, medical therapy was limited by worsening renal function and hyperkalemia. He was also not a candidate for established device therapies such as mitral clip or cardiac resynchronization therapy. After a discussion with the patient about his limited options, we offered CCM implantation to improve his HF symptoms and functional capacity.

##### Clinical outcome

After implantation, the patient had a significant and sustained reduction in diuretic requirements, an improvement from NYHA Class III to NYHA Class II, and a notable decrease in NT-proBNP levels despite persistently poor renal function. His daily diuretic requirement decreased from torsemide 60 mg three times per day to 20 mg once daily over the following months. He has remained hospitalization-free for 18 months post-CCM.

**Figure 3:**
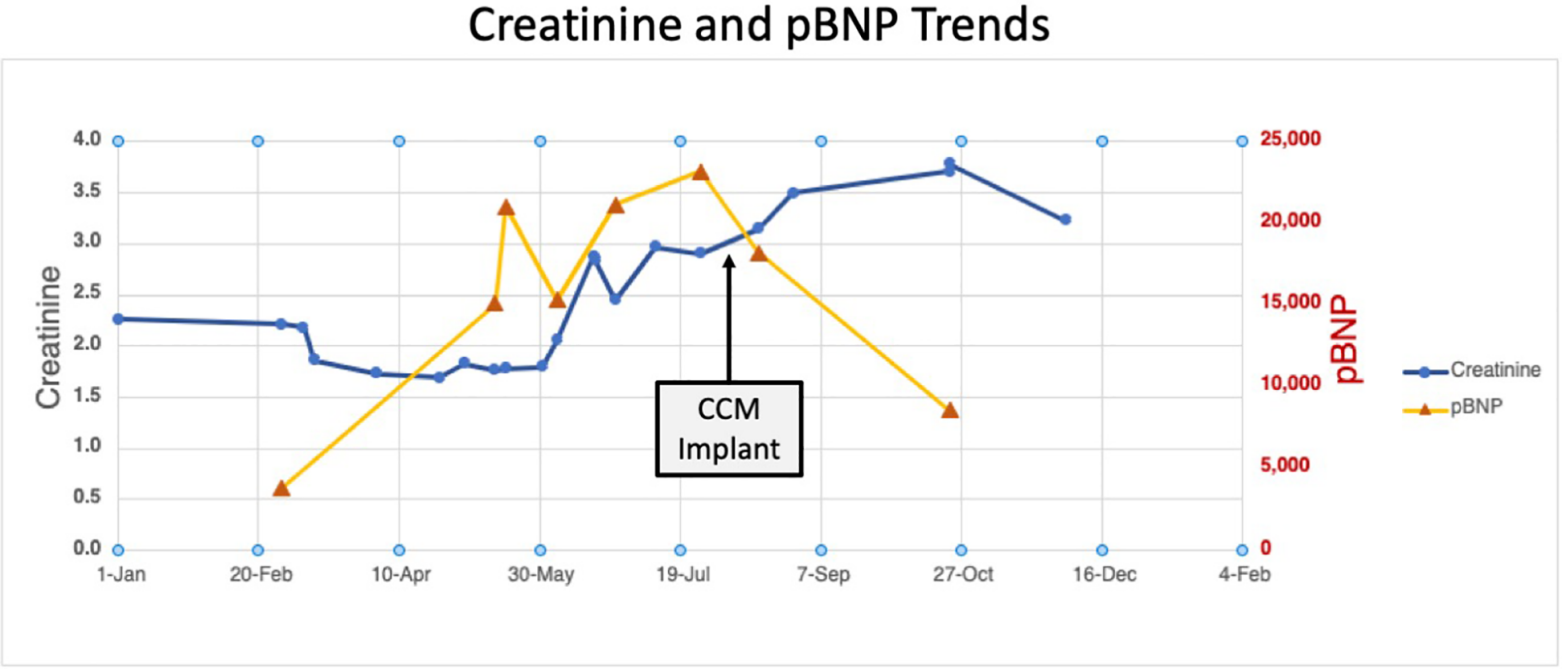
Case 1—creatinine and proBNP trends before and after CCM implant.

Figure 3 shows creatinine and NT-proBNP trends before and after the intervention.

#### Case 2 Phenoprofile II: GDMT intolerance due to hypotension—leveraging BAT in symptomatic NYHA Class III HFrEF

##### Clinical scenario

A 79-year-old man with a history of HfrEF secondary to ischemic cardiomyopathy and severe MR, who had a CRT-D device implanted 4 years ago, was referred to the structural heart disease clinic by his primary cardiologist for evaluation of severe MR with the symptoms of exertional dyspnea and dizziness. He was initially considered for MitraClip evaluation, but suboptimal valve anatomy precluded transcatheter edge-to-edge repair, leading to a referral to the HF clinic. At the time of assessment, his echocardiogram showed an LVEF of 20% and LVEDD of 7.2 cm, and his proBNP was 1,453 pg/ml. He was initially classified as NYHA Class III and was unable to tolerate standard GDMT regimens due to hypotension and dizziness.

##### HF device therapy selected

Given his severely reduced LVEF and proBNP below 1,600 pg/ml with intolerance to GDMT escalation and no option for other device-based therapies or for HRT due to advanced age, the decision was made to proceed with BAT implantation for symptomatic HF improvement and reduction in HFH risk.

##### Clinical outcome

After BAT implantation, settings were gradually titrated during electrophysiology clinic visits over the next 3 months following the procedure. This approach led to a modest improvement in his dyspnea and a significant reduction in dizziness, allowing further optimization of his GDMT. By 9 months post-implantation, the patient's symptoms had improved to NYHA Class II, and his GDMT regimen was successfully advanced to more tolerant and optimized GDMT regimens.

#### Case 3 Phenoprofile III: GDMT unresponsiveness in a potential HRT candidate—a hypothetical case of HFrEF managed with AccuCinch TLVR

##### Clinical scenario

A 46-year-old man with a history of HFrEF due to non-ischemic cardiomyopathy had been treated with optimal GDMT and later received an ICD due to persistent left ventricular dysfunction. He reported being able to walk 5–6 blocks, though 2 years earlier, he was actively participating in recreational sports. Cardiopulmonary exercise testing (CPET) showed a peak oxygen uptake (pVO2) of approximately 14 ml/kg/min and a VE/VCO2 ratio of 34. Despite some high-risk features, he maintained reasonable exercise tolerance, had no recent hospitalizations, and continued to tolerate target doses of quadruple GDMT. On echocardiogram, his LVEF was 25%, with an LVEDD of 6.5 cm, and his symptoms were consistent with NYHA Class III.

##### HF device therapy selected

Given that he had several high-risk features but a limited but reasonable quality of life and no recurrent HFH, he was deemed to be too early in his course for HRT, and his options were to continue GDMT or to receive a novel device-based therapy for HF. After an in-depth discussion about his therapeutic options, he agreed to be screened for the CORCINCH-HF trial where he would be randomized to receive the AccuCinch TLVR vs. ongoing medical therapy. This would serve as an intermediate strategy while preserving his long-term eligibility for HRT. The patient met all anatomical and clinical criteria and was successfully randomized into the trial.

##### Clinical outcome

Following uneventful randomization, he has continued follow-up in the HF clinic and is undergoing serial assessments per the trial protocol. In the meantime, his eligibility remains unchanged for LVAD or transplant if he were to deteriorate clinically.

#### Case 4 Phenoprofile IV: HFrEF with GDMT unresponsiveness in an initially non-HRT candidate—bridging to durable LVAD with CCM

##### Clinical scenario

A 71-year-old man with HFrEF due to ischemic cardiomyopathy and multi-vessel coronary disease, which was deemed unrevascularizable due to the absence of myocardial viability, had previously undergone ICD placement and had a narrow QRS duration. Despite multiple attempts to escalate GDMT, he experienced recurrent heart failure hospitalizations, including one episode of cardiogenic shock that required intubation and intra-aortic balloon pump placement. Even with the use of CardioMEMS to guide his medical therapy, he continued to suffer from NYHA Class III symptoms, necessitating high daily doses of diuretics. His GDMT was further limited by hypotension and dizziness, leading to a referral for LVAD evaluation. However, the work-up revealed severe bilateral carotid artery stenosis, with near-total occlusion of the left common carotid artery, making him an unsuitable candidate for HRT.

##### HF device therapy selected

On repeat assessment in the HF clinic, his LVEF remained at 30%, with an LVEDD of 5.5 cm, and he continued to have NYHA Class III symptoms. Given his complex condition at that time, he was ineligible for both carotid artery surgery and heart replacement therapies. After discussing his options, he was offered CCM as a palliative therapy to improve the symptoms of his stage D HFrEF based on the approved criteria for this device. He agreed and his implant was uneventful.

##### Clinical outcome

Within 6 weeks of the CCM implantation, he experienced significant symptomatic improvement, and over the next year, he remained stable with no rehospitalizations. His symptoms improved to NYHA Class II. Three months later, given his clinical improvement, he successfully underwent transcarotid artery revascularization without complications. However, 9 months later, he was readmitted with ADHF deteriorating into cardiogenic shock requiring intra-aortic balloon pump placement. With his carotid disease now revascularized, he was re-evaluated and deemed an appropriate candidate for LVAD implantation. He underwent a successful LVAD implantation during the same admission, with the CCM device being removed concurrently. The patient was discharged uneventfully and continued to be followed up in the HF clinic. Hence, in this case, CCM effectively served a “bridge-to-candidacy” role which could be considered in similar cases.

**Figure 4:**
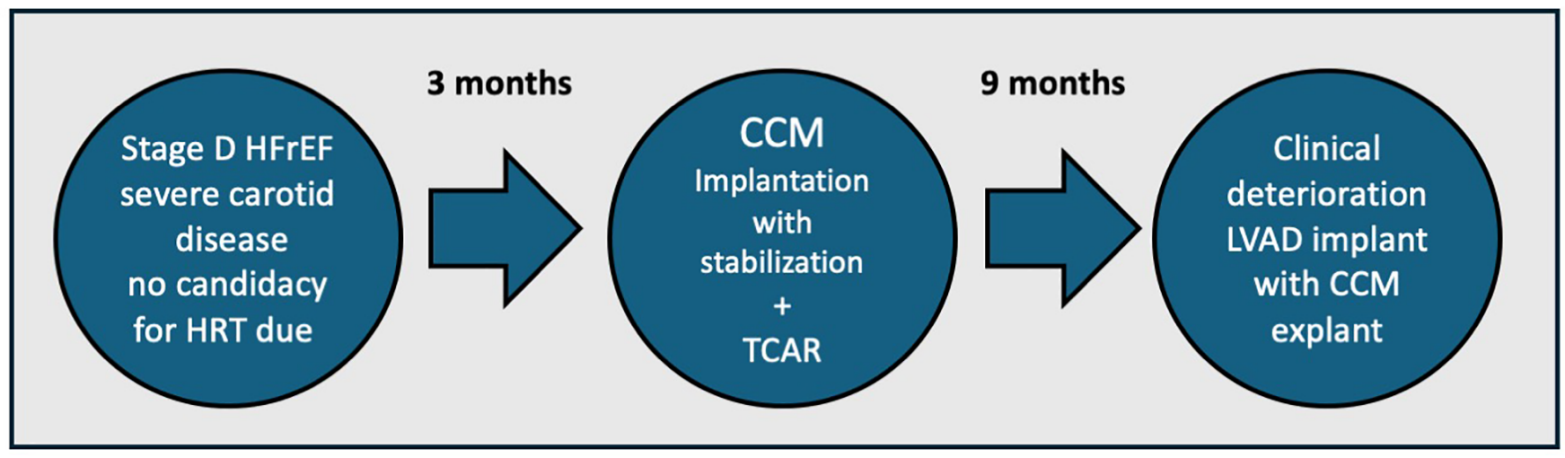
Case 4—clinical trajectory with tandem use of CCM and LVAD.

Figure 4 shows the clinical trajectory with tandem use of CCM and LVAD.

#### Case 5 Phenoprofile II: GDMT intolerant and not an HRT candidate treated with MitraClip

##### Clinical scenario

A 77-year-old male with a history of HFrEF due to non-ischemic cardiomyopathy, severe functional MR, and CKD IV (baseline Cr, 2.4 mg/dl) had recurrent admissions for ADHF and NYHA Class III baseline symptoms. The initial echocardiogram demonstrated biventricular dysfunction with LVEF of 31%, LVEDD of 4.8 cm, and severe MR with NT-proBNP of 53,448 pg/ml. He was treated with diuretics and discharged on low doses of GDMT but experienced breakthrough decompensation leading to subsequent rehospitalization 1 month later. His renal function worsened (Cr 4.1 mg/dl), resulting in marked hyperkalemia, and he was found to have a progression of first-degree AV block with symptomatic hypotension. As a result, he remained only on a low-dose beta-locker, hydralazine, and isosorbide dinitrate with borderline blood pressure.

##### HF device therapy selected

Given the intolerance to standard GDMT, poor functional capacity, and persistently reduced LVEF with severe MR, he was considered for HRT and deemed a poor candidate due to advanced age and profound deconditioning. He underwent MitraClip evaluation with the structural heart team and was offered this intervention for HF improvement and reduction of HFH and mortality risk.

##### Clinical outcome

**Figure 5:**
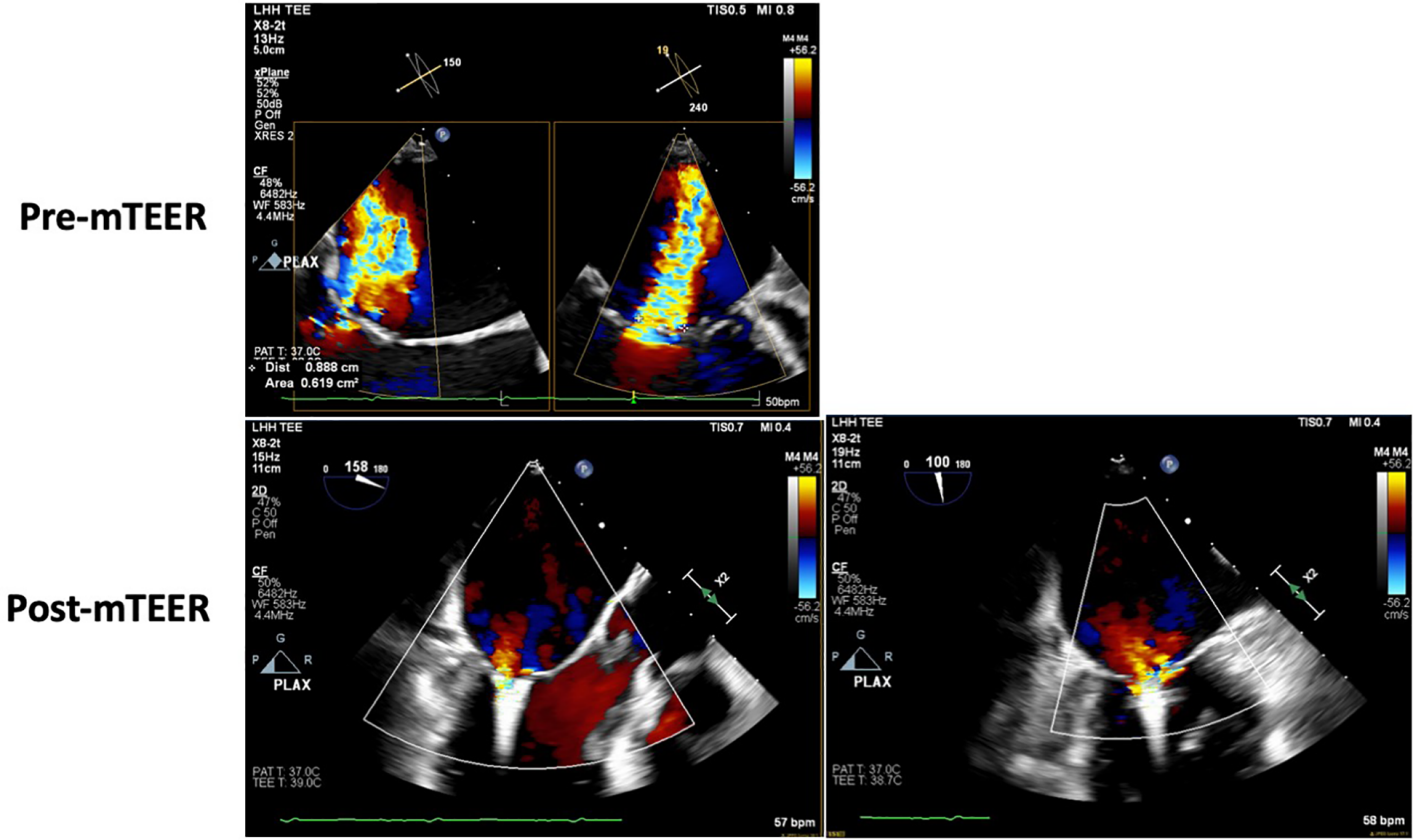
Case 5—transesophageal echocardiogram showing pre- and post-mitral TEER severity of mitral regurgitation.

He underwent successful transcatheter mitral edge-to-edge repair with a MitraClip, resulting in mild-to-moderate MR (Figure 5). He was discharged and tolerated low-dose ARNI and SGLT2i with recovery to baseline renal function. A follow-up TTE performed 1 week after the procedure showed an LVEF of 35% with trace MR. Four weeks status-post MitralClip, he reported objective improvement in dyspnea and was able to participate in physical therapy, walking, or biking for 20 min at a time. He continues to be closely monitored by the heart failure clinic, tolerating escalation to maximal dose ARNI and the addition of low-dose beta-blocker. He has not had an HFH for over 9 months at the time of this report.

## Conclusion

The landscape of HF treatment is rapidly evolving, with numerous novel devices emerging as therapeutic options. While some devices have already been approved for commercial use, others are still in the pivotal trial phase. These innovations, ranging from electrical-based therapies to devices designed to modify cardiac structure, offer new possibilities for patient care. Hence, our approach focuses on distinct HF phenoprofiles based on response to GDMT and eligibility for HRT, aiming to match these novel technologies with the patients likely to derive the most benefit. As new evidence emerges, it will refine our treatment paradigms, expanding options for a broader range of HF patients.

## Glossary

ACC: American College of Cardiology
ACEI: angiotensin-converting enzyme inhibitors
ADHF: acute decompensated heart failure
AHI: apnea–hypopnea index
ARB: angiotensin receptor blockers
ARNI: angiotensin receptor neprilysin inhibitors
BAT: baroreflex activation therapy
CCM: cardiac contractility modulation
CHAMP-HF: Change the Management of Patients with Heart Failure
CKD: chronic kidney disease
CRT: cardiac resynchronization therapy
CRT-D: cardiac resynchronization therapy-defibrillator
CSA: central sleep apnea
ESC: European Society of Cardiology
FDA: Food and Drug Administration
GDMT: guideline-directed medical therapy
GFR: glomerular filtration rate
HF: heart failure
HFH: heart failure hospitalization
HFpEF: heart failure with preserved ejection fraction
HFrEF: heart failure with reduced ejection fraction
HFSA: Heart Failure Society of America
HRT: heart replacement therapies
ICD: implantable cardioverter defibrillator
IMPROVE-HF: Registry to Improve the Use of Evidence-Based Heart Failure Therapies in the Outpatient Setting
LV: left ventricle
LVAD: left ventricular assist device
LVEDD: left ventricular end-diastolic diameter
LVEDV: left ventricular end-diastolic volume
LVEF: left ventricular ejection fraction
MAGGIC: Meta-analysis Global Group in Chronic Heart Failure
MLWHFQ: Minnesota Living with Heart Failure Questionnaire
MR: mitral regurgitation
MRA: mineralocorticoid receptor antagonists
MRI: magnetic resonance imaging
NT-proBNP: N-terminal pro-B-type natriuretic peptide
NYHA: New York Heart Association
PAP: pulmonary artery pressure
PARADIGM-HF: Prospective Comparison of ARNI with ACEI to Determine Impact on Global Mortality and Morbidity in Heart Failure
pVO2: peak oxygen uptake
SGLT2i: sodium–glucose cotransporter-2 inhibitors
TR: tricuspid regurgitation
TLVR: transcatheter left ventricular restoration
VE/VCO2: ventilation-to-carbon dioxide production ratio.
